# Consideration of Psychosocial Factors in Acute Low Back Pain by Physical Therapists

**DOI:** 10.3390/jcm12113865

**Published:** 2023-06-05

**Authors:** Emilia Otero-Ketterer, Cecilia Peñacoba-Puente, Ricardo Ortega-Santiago, Fernando Galán-del-Río, Juan Antonio Valera-Calero

**Affiliations:** 1 Escuela Internacional de Doctorado, Universidad Rey Juan Carlos, 28922 Alcorcón, Spain; eoteroke@mutuauniversal.net; 2 Physiotherapy Department, Mutua Universal Mugenat, 28801 Alcalá de Henares, Spain; 3 Department of Psychology, Universidad Rey Juan Carlos, 28922 Alcorcón, Spain; cecilia.penacoba@urjc.es; 4 Cátedra Institucional en Docencia, Clínica e Investigación en Fisioterapia: Terapia Manual, Punción Seca y Ejercicio Terapéutico, Universidad Rey Juan Carlos, 28922 Alcorcón, Spain; 5 Department of Physical Therapy, Occupational Therapy, Physical Medicine and Rehabilitation, Universidad Rey Juan Carlos, 28922 Alcorcón, Spain; fernando.galandel@urjc.es; 6 Department of Radiology, Rehabilitation and Physiotherapy, Faculty of Nursery, Physiotherapy and Podiatry, Universidad Complutense de Madrid, 28040 Madrid, Spain; juavaler@ucm.es; 7 Grupo InPhysio, Instituto de Investigación Sanitaria del Hospital Clínico San Carlos (IdISSC), 28040 Madrid, Spain

**Keywords:** biopsychosocial models, low back pain, physiotherapy, psychosocial factors, survey

## Abstract

Clinical guidelines consistently recommend screening psychosocial (PS) factors in patients with low back pain (LBP), regardless of its mechanical nature, as recognized contributors to pain chronicity. However, the ability of physiotherapists (PTs) in identifying these factors remains controversial. This study aimed to assess the current identification of psychosocial risk factors by physical therapists (PTs) and which characteristics of PTs are associated with the identification of the main risk for chronicity (physical or psychosocial). A cross-sectional descriptive study surveying Spanish PTs in public and private health services was conducted, including questions on PT characteristics and three low back pain (LBP) patient vignettes with different biopsychosocial (BPS) clinical presentations. From 484 respondents, the majority of PTs agreed regarding the main risk for chronicity for each vignette (PS 95.7% for vignette A, PS and physical 83.5% for vignette B and PS 66% for vignette C). Female PTs were more likely to rate psychosocial compared with males (*p* < 0.05). PTs with higher levels of social and emotional intelligence (both, *p* < 0.05) were more likely to identify the main risk for chronicity. However, only gender and social information processing for vignette A (*p* = 0.024) and emotional clarity for vignette B (*p* = 0.006) were able to predict the identification of psychosocial and physical risk, respectively. The main risk for chronicity was correctly identified by a large majority of PTs through patient vignettes. Gender, social and emotional intelligence played a relevant role in the recognition of psychosocial risk and biopsychosocial factors.

## 1. Introduction

The paradoxical increase in low back pain (LBP) disability during the application of a tissue-centered biomedical approach generated the proposal of the biopsychosocial (BPS) model [1,2,3,4,5]. This model was based on the recognition of the biological, psychological and social interactions within the clinical evaluation and treatment of the disease [6], being adopted by the World Health Organization in the International Classification of Functioning, Disability and Health in 2000 [7] up to the present. Since its conception, the BPS model distinguished low back pain from low back disability according to the presence of patients with similar findings in imaging tests of the lumbar spine and with disparate levels of disability due to pain [6]. 

Although approximately 90% of cases of LBP are not associated with a precise pathoanatomical diagnosis [8], its inexistence cannot be stated [9]. This condition has been commonly referred to as non-specific LBP [10] and currently termed nociplastic, referring to the fact that there may be altered nociception [11,12]. Therefore, the differentiation made by the BPS model between the source of pain and the disability it generates is still relevant. Biological, psychological and social aspects, context, and classical and operant behavioral conditioning [13,14] shape, to a greater or lesser extent, the individual experience of pain [15,16]. Thus, psychosocial (PS) factors play a role in the clinical presentation of patients and are known predictors of the development and maintenance of chronic pain [17,18,19,20], being even more determinant aspects than somatosensory and motor alterations in LBP individuals [21,22].

Therefore, early identification of patients at risk of poor prognosis for recovery is critical to target modifiable factors and minimize the risk of chronicity of symptoms [23]. Clinical guidelines consistently recommend screening for PS factors in all patients with LBP, regardless of its mechanical nature [24,25,26], using validated patient-reported outcome measures in combination with clinical judgment [27,28]. Research reflects contradictory findings between the poor ability of physical therapists (PTs) to identify individual constructs such as fear avoidance, kinesiophobia, or emotional distress [29,30,31] as well as to allocate LBP patients into risk stratification groups [32] and those suggesting their adequate intuition in this regard [33,34,35].

Despite the clinical relevance of PT ability to detect PS factors in LBP patients, studies on this issue related to the sociodemographic or professional characteristics of PTs are scarce, and to the best of our knowledge, there are no studies carried out in Spain. Furthermore, although emotional and social intelligence are valuable assets for health care professionals [36,37] as they deal with people who are under emotional stress [38], being increasingly important for good physical therapist–patient interaction [39], there are no known studies assessing their influence on the detection of PS factors in LBP. A better understanding in this regard can lead to its inclusion in the development of educational training aimed at the identification and management of the psychosocial status of patients.

Therefore, the objective of this study is to assess the current identification of psychosocial risk factors by PTs. In addition, we aim to explore whether sociodemographic (age and gender), professional (years of clinical experience, work setting and postgraduate training in PS factors) and attitudinal aspects related to social and emotional intelligence of PTs are associated with the identification of the main risk for chronicity (physical or psychosocial).

## 2. Materials and Methods

This is a descriptive, cross-sectional study using an online survey. The Checklist for Reporting Results of Internet E-Surveys (CHERRIES) has been followed [40] and the study protocol was revised and approved by the Clinical Ethics Committee of Hospital Clínico San Carlos (ID: 21/257). An informed consent form was drafted containing information about the expected time of the survey (approximately 20 min), data storage, researchers’ information and the study aim. After reading and signing the informed consent form, as well as meeting the inclusion criteria (e.g., being a regulated PT and having treated at least one case of acute LBP in the last year), participants were eligible. No incentives were offered to provide the survey results.

### 2.1. Study Population

The survey was based on questions about the characteristics of PTs in terms of their identification of the main risk for chronicity (physical or psychosocial) in three patient vignettes (case scenarios) with acute LBP. Thus, PTs in Public Health Service, Mutual Health Insurance Companies collaborating with the Public Health Service, and private practice from multiple regions of Spain were invited to participate through online announcements during 2020. To meet sample size requirements and above the recommendation of collecting 10 events (cases) for each predictor variable [41] (i.e., 70 respondents considering the 7 predictor variables in our study), we aimed to obtain at least 100 cases for each physical therapy work setting (a total of 300 cases) in order to provide the most adequate descriptive information on the identification of PS factors.

### 2.2. Study Development

The open survey instrument was approached in three phases. In the first stage, the patient vignettes were constructed reflecting different BPS clinical presentations: vignette A (high psychosocial risk); vignette B (high physical risk); and vignette C (high physical and psychosocial risk). Information on clinical history, symptoms, relevant physical findings and PS status was included. These case scenarios were validated by a group of national experts, selected by the authors on the basis of their clinical and research trajectory. Two expert psychologists in pain, as well as two rehabilitation physicians and three PTs with expertise in the treatment of musculoskeletal pain rated the main risk for chronicity in each vignette. There was agreement from all experts on the rating except two experts who commented on a higher physical risk in vignette A, so minor corrections were made to that vignette to aid differentiation with the main PS risk. Then, the principal investigator developed a draft released on Google Forms (https://docs.google.com/forms/) (accessed on 12 September 2022) that included the questions (one per page) on PT characteristics and patient vignettes, which was tested with a small sample of 15 participants for potential comprehension, usability, and technical functionality issues prior to initiating the research. Participants had not enabled the option for review and change their answers. As no problems were found, the final version was published openly to initiate data collection. 

### 2.3. Outcome Variables

#### 2.3.1. Screening Questionnaire for Psychosocial Factors in LBP

Following the procedure described above, the ad hoc instrument relating to the detection of PS factors in LBP patients consisted of three items (patient vignettes) with the distribution: vignette A (high psychosocial risk); vignette B (high physical risk); and vignette C (high physical and psychosocial risk). In each of these, the physical therapist was asked to answer the next question: “Of the following types of factors, which one contributes most to this patient’s risk of chronicity? Physical or Psychosocial”. 

#### 2.3.2. Sociodemographic and Professional Variables

Sociodemographic factors (age and gender) and professional factors (years of clinical experience treating musculoskeletal disorders, work setting and postgraduate training in PS factors) were assessed.

#### 2.3.3. Emotional and Social Intelligence

To assess social intelligence, understood as the ability to communicate and form relationships with empathy and assertiveness, we used the Social Intelligence Questionnaire [42] which is composed of 34 items, with a five-point Likert scale. The scale allows the assessment of five dimensions of social intelligence: self-monitoring, social sensitivity, social information processing, social skills and social awareness (Cronbach’s alpha = 0.78–0.82) [42].

The Spanish version [43] of the Trait Meta Mood Scale (TMMS-24) was used for assessing emotional intelligence, which has shown high internal consistency and satisfactory test–retest reliability [44]. This scale is composed of 24 items, with a five-point Likert scale and allows the assessment of three dimensions of emotional intelligence: Emotional attention, clarity and repair (Cronbach’s alpha = 0.80–0.86) (see Supplementary File S1 for checking the survey questionnaire form) [44].

### 2.4. Statistical Analysis

Data from Google Forms were transferred to the SPSS statistical package (version 27.0) for Mac OS to be analyzed. First, a Kolmogorov–Smirnov test was used to determine the normal distribution of the data. Later, all PT characteristics were analyzed using descriptive statistics and crosstabulations. First, bivariate analyzes were carried out. Specifically, differences in the rating of the main chronicity risk were explored using the unpaired Student *t*-test for the quantitative variables and Pearson chi-square test for the qualitative variables. Considering the variables that had been significant in the bivariate analyzes, a stepwise multiple regression was calculated to assess which variable (sociodemographic, professional and social and emotional intelligence) best predicts the identification of the main risk factor for chronicity [45]. All statistical analyses were conducted setting a 95% confidence level, and a significance level of *p* < 0.05 to be considered statistically significant.

## 3. Results

Four hundred and eighty-four PTs completed the survey (67.1% female; age: 35 ± 8.2 years). Only 4 of 17 Official Associations of Physiotherapy did not publish the survey. Given that an unknown number of PTs viewed the announcements on the web pages and social media of their Official Associations, it was not possible to calculate the response rate obtained. Respondents’ sociodemographic, professional, and attitudinal characteristics are described in Table 1, Table 2 and Table 3, respectively, for the total study population and for the number of PTs who responded correctly to the main risk factor for chronicity in each vignette.

Table 4 reports agreement in the majority of PTs, with the main risk for chronicity stated for each of the patient vignettes—95.7% (n = 464) and 83.5% (n = 405) stated the main risk as psychosocial and physical for vignettes A and B, respectively, and 66% (n = 320) stating psychosocial risk for the vignette C.

The bivariate analyzes showed that in none of the three case scenarios were ratings of the main risk for chronicity associated with work setting or postgraduate training in PS factors (Table 2 and Table 3). However, there was a significant association on the gender variable for vignettes A and C, in which female PTs were more likely to rate the main risk of chronicity as psychosocial (ꭓ^2^ = 14.7, *p* < 0.001 and ꭓ^2^ = 3.8, *p* = 0.049, respectively), with no statistical difference found for vignette B (ꭓ^2^ = 0.78, *p* = 0.38). In addition, age and years of clinical experience in treating musculoskeletal disorders was associated with the rating for vignette C, in which older and more experienced PTs were more likely to state physical risk over psychosocial risk as the main risk factor (mean 36.6 ± 9, *p* = 0.016 and mean 13.1 ± 7.9, *p* = 0.004, respectively). Furthermore, there were significant associations on certain aspects of emotional and social intelligence for vignettes A and B, in which PTs with higher emotional attention, social sensitivity and social information processing were more likely to rate the main risk in vignette A as psychosocial (mean 27.1, SD ± 6, *p* = 0.012; mean 21.4, SD ± 3.6, *p* < 0.01 and mean 25.1, SD ± 4, *p* = 0.023, respectively), as well as PTs with higher emotional clarity and social information processing were more likely to rate the main risk in vignette B as physical (mean 30.2, SD ± 5.6, *p* = 0.006 and mean 25.2, SD ± 4, *p* = 0.018, respectively) (Table 2 and Table 3).

The results of the logistic regression analysis showed that only some variables, social information processing (B = −0.117, *p* = 0.024, Exp (B) = 0.89) and gender (B = −1.717, *p* = 0.001, Exp (B) = 0.180) for vignette A (R2 Cox y Snell = 0.37, *p* = 0.024) as well as emotional clarity (B = 0.058, *p* = 0.006, Exp (B) = 1.059) for vignette B (R2 Cox y Snell = 0.17, *p* = 0.006), remained significantly associated with the identification of the main risk for chronicity, psychosocial and physical, respectively, after the inclusion of gender as a covariate.

## 4. Discussion

This study is the first nationwide survey in Spain exploring the identification of PS risk factors by PTs working in the musculoskeletal field, framed within the adoption of the biopsychosocial model in patients with LBP. In addition, we analyzed whether different sociodemographic, professional and attitudinal characteristics of these health care professionals were associated with the detection of the main risk for chronicity, and which variables best predicted their identification, providing insights into the aspects that influence their recognition. Overall, the identification of the main risk for chronicity was properly carried out by a large majority of PTs through LBP patient vignettes. 

The ability of PTs to rate chronicity risk according to PS aspects has been reported to be adequate for high and low risk cases, but not for moderate risk cases in a survey study also based on LBP patient vignettes [46]. Correct estimates of BPS risk of chronicity by PTs were also reported by comparing clinical assessments of actual LBP patients, with respect to patient responses on the Orebro Musculoskeletal Pain Questionnaire (OMPQ) [33,34], as well as from PTs who followed a BPS training program (35). However, Wassinger et al. recently reported slight agreement between the PTs’ ratings and OMPQ during the evaluation of musculoskeletal conditions [47]. Other studies have described difficulties of PTs in identifying individual constructs such as depression, fear avoidance and pain catastrophizing in patients with LBP [27,29,31,32]. Moreover, Beales et al. found inconsistent correlations for PT estimates of each of the OMPQ domains [33], although this inconsistency may have been influenced by the fact that such constructs were not measured using the established assessment tools and their cut-off points [31]. All these conflicts in findings may be partly explained by the methodology, either in relation to the use of actual patients versus patient vignettes or the use of different assessment tools. However, underreporting by some of these authors of PT characteristics that could have influenced their results has also been detected, such as those characteristics mentioned above [29,32,34,35]. 

Among the characteristics of PTs associated with the identification of PS factors, in our sample, female PTs better identified the main PS risk in patient vignette A than male PTs. In addition, female PTs were more likely to rate the main risk for chronicity in patient vignette C as psychosocial versus physical than male PTs and estimated the main physical risk of patient vignette B similarly to male PTs. These results suggest that the ability of female PTs was not biased toward the PS component, being better at identifying PS chronicity risk than male PTs. These findings support the results presented by a meta-analytic review showing that female physicians address psychosocial and emotional information about their patients during medical visits to a greater extent than male physicians, having found no differences in the handling of biomedical information [48]. 

Several aspects of the PTs’ social and emotional intelligence influenced the correct discrimination between the main physical and psychosocial risk for vignettes A and B. However, stepwise multiple regression analysis showed that only social information processing (component of social intelligence relating to how people develop and handle relationship information) and emotional clarity (component of emotional intelligence that allows us to integrate what we feel into our thinking and to be able to consider the complexity of emotional changes) remained significantly associated with their recognition. Emotional and social intelligence is the ability to understand and manage emotions in oneself and others [36]. Although the importance of these skills in meeting new communication challenges, transfer of knowledge available for clinical practice and patients’ education, among others, has recently been described [37], studies specifically analyzing how social and emotional intelligence influence the implementation of the BPS approach are lacking. PTs have traditionally focused on structural/biomechanical care, but the need for interventions directed at the BPS components of the individual experience of pain is becoming increasingly relevant [49] as they are ideally situated to identify barriers to recovery and reduce pain experiences by educating and addressing patients’ dysfunctional beliefs about pain [50,51,52]. Since there are ways to develop the social and emotional intelligence skills of health care professionals [53], incorporating these aspects in the development of interventions and trainings aimed at the BPS approach of PTs may also be useful to overcome the challenge of the traditional roles involved [36,54]. 

Formal education [55,56] as well as clinical experience and on-the-job learning [57] make up expertise in physiotherapy. We asked whether postgraduate training in PS aspects, older and more experienced PTs and work setting influenced recognition of PS factors, finding no statistically significant differences except for older and more experienced PTs, that were more likely to report main physical risk in patient vignette C. This significant finding could reflect some biomedical bias for more experienced PTs, although this trend did not imply a worse identification of PS risk in patient vignette A, similar to that reported by other authors [31]. In addition, although only one-third of PTs in our sample reported having received such PS training (29.5%, n = 143), our results could reflect the difficulty of changing behaviors in clinical practice after BPS training, reported by other studies [58,59,60]. On the other hand, it has been reported that PTs working in community orthopedic settings held attitudes toward LBP (HC-Pairs questionnaire) better aligned with best practice evidence than non-community orthopedic PTs [61]. The difference with our findings can be explained by the fact that the work settings considered in our study were all clinical environments.

### Limitations

This study has several limitations. First, the vignettes tend not to represent a real clinical situation and also, our findings depended on participants’ self-reports, so we cannot infer our results to the identification of the main risk for chronicity by PTs in their daily clinical practice. However, some authors have noted the tendency of studies employing “real patients” to show favorable results for clinicians’ assessment of chronicity risk and/or identification of psychosocial factors compared to assessment instruments versus those employing videos or vignettes [33]. Variations in health care delivery by PTs toward LBP patients with PS risk factors are widespread. Such variations are driven by the pain-related attitudes and beliefs of these professionals compatible with a biomedical approach, sometimes stigmatizing these patients and feeling unprepared to treat them, resulting in negative and unhelpful consequences in the therapeutic encounter [62,63]. Obtaining useful information on the identification of PS chronicity risk for PTs within health care systems is a challenge, even more so when the situations to be assessed are complex or confront the traditional roles involved in the profession. Several validation studies have found that the use of vignettes reflects the stated intentions and behaviors of health professionals more closely than data extracted from recordings of actual consultations [64,65,66]. While both methods have strengths and weaknesses, well-designed vignette studies may have advantages in certain scenarios [67]. To this end, several steps were taken in the present study to address the quality criteria, including a description of the steps followed for vignette development, validation by an expert panel, and pretest, to explore participants’ likely judgments by presenting a series of hypothetical but realistic clinical scenarios, and to generate inferences about the relationships between the nature of each vignette and the PTs’ subsequent responses to them [68]. Moreover, although the case scenarios shown in this study do not represent the wide variety of PS aspects that may be present in patients with LBP, and focused more on the psychological emotional and social components of pain, recent evidence has highlighted less attention to such aspects when adopting the biopsychosocial model in physical therapy settings compared to cognitive and behavioral ones [69], so an analysis focused on how their identification by PTs is performed is currently relevant. Additionally, in the present study we explicitly asked the participants to select between the main risk of chronicity, physical or psychosocial. Although this could have been oriented towards one of these two response options, all the scenarios of LBP cases shown presented a physical component to a greater or lesser degree, which allowed us to analyze the PTs’ evaluation of its relevance with respect to chronicity and the existence of a possible bias towards the physical aspects of pain, even though the PS component was present (vignette A). In addition, a third vignette (vignette C) was presented, with a high risk of both physical and psychosocial chronicity, which allowed us to contrast the possible existence of biases in the identification of the physical and psychosocial aspects in the responses shown to vignettes A and B. Second, given that in this study it was not possible to calculate the response rate and that respondents could be interested in PS factors, PTs’ abilities to identify PS risk may be biased. However, even if non-response was not random, in survey studies, non-response does not appear to generate significant response bias [70]. Finally, the participants in our sample had on average a high level of clinical experience, so we cannot generalize the results to more recently qualified PTs.

## 5. Conclusions

The main risk for chronicity was correctly identified by most of PTs. Our findings suggest that female PTs were better at identifying PS chronicity risk than male PTs and several aspects of the PTs’ social and emotional intelligence also influenced the correct distinction between the main physical and psychosocial risk. However, only gender and social information processing as well as emotional clarity were statistically significant in predicting accurate identification of the main risk of psychosocial and physical chronicity, respectively. Future research may analyze whether incorporating these attitudinal aspects into training strategies improves PT recognition in clinical practice and patient outcomes.

## Figures and Tables

**Table 1:** Sociodemographic characteristics for the total study population of PTs (n = 484) and their responses on the main risk factor for chronicity of each vignette.

		Identification of the Main Risk Factor for Chronicity						
	Total Study Population (n = 484)	Vignette A	*p* Value	Vignette B	*p* Value	Vignette C		*p* Value
		Psychosocial (n = 464)		Physical (n = 405)		Psychosocial (n = 320)	Physical (n = 165)	
Age (years), mean (SD)	35 (8.2)	35.3 (8.3)	0.42	35.2 (8.2)	0.68	34.6 (7.8)	36.6 (9)	0.016
Gender, n (%)			<0.01		0.38			0.049
Females	325 (67.1)	319 (68.8)		268 (66.2)		224 (70)	101 (61.2)	
Males	159 (32.9)	145 (31.3)		137 (33.8)		96 (30)	64 (38.8)	

**Table 2:** Professional characteristics for the total study population of PTs (n = 484) and their responses on the main risk factor for chronicity of each vignette.

	Identification of the Main Risk Factor for Chronicity							
	Total Study Population (n = 484)	Vignette A	*p* Value	Vignette B	*p* Value	Vignette C		*p* Value
		Psychosocial (n = 464)		Physical (n = 405)		Psychosocial (n = 320)	Physical (n = 165)	
Experience (years), mean (SD)	12 (7.4)	11.8 (7.5)	0.21	11.8 (7.3)	0.6	11.1 (7.1)	13.1 (7.9)	0.004
Work setting, n (%)			0.65		0.3			0.71
Private practice	272 (56.2)	259 (55.8)		222 (54.8)		178 (55.6)	94 (57)	
Public Health Service	109 (22.5)	106 (22.8)		96 (23.7)		70 (21.9)	39 (23.6)	
Insurance Companies	103 (21.3)	99 (21.3)		87 (21.5)		72 (22.5)	32 (19.4)	
Postgraduate training in PS factors, n (%)			0.07		0.64			0.2
Yes	143 (29.5)	134 (28.9)		122 (30.1)		89 (27.8)	55 (33.3)	
No	341 (70.5)	330 (71.1)		283 (69.9)		231 (72.2)	110 (66.7)	

**Table 3:** Attitudinal characteristics for the total study population of PTs (n = 484) and their responses on the main risk factor for chronicity of each vignette.

	Total Study Population (n = 484)	Vignette A	*p* Value	Vignette B	*p* Value	Vignette C		*p* Value
		Psychosocial (n = 464)		Physical (n = 405)		Psychosocial (n = 320)	Physical (n = 165)	
Emotional Intelligence	87 (12.6)	87.7 (12.1)	0.11	87.9 (11.7)	0.13	87.5 (12.1)	87.6 (12.9)	0.92
Emotional Attention	27.1 (6.1)	27.1 (6)	0.01	26.9 (6)	0.65	27.1 (6)	26.6 (6.2)	0.34
Emotional Clarity	29.5 (5.8)	29.9 (5.5)	0.78	30.2 (5.6)	<0.01	29.8 (5.8)	30 (5.5)	0.60
Emotional Repair	30.4 (5.7)	30.7 (5.5)	0.63	30.7 (5.4)	0.30	30.5 (5.5)	30.9 (5.6)	0.47
Social Intelligence	123.7 (15.1)	124.4 (14.8)	0.04	124.6 (14.8)	0.11	124.2 (14.9)	124 (15.3)	0.84
Self Monitoring	25.8 (4)	25.9 (3.9)	0.07	26 (4)	0.24	26 (3.8)	25.7 (4.2)	0.33
Social Sensitivity	21.3 (3.7)	21.4 (3.6)	0.01	21.4 (3.6)	0.23	21.3 (3.5)	21.2 (4)	0.86
Social Information Processing	25 (4.2)	25.1 (4)	0.02	25.2 (4)	0.01	25 (4.1)	25 (4.2)	0.89
Social Skills	25.8 (4.8)	26.1 (4.7)	0.28	26.1 (4.7)	0.24	26 (4.9)	25.9 (4.5)	0.77
Social Awareness	25.8 (4.3)	25.9 (4.1)	0.78	25.9 (4.2)	0.92	25.8 (4.2)	26.1 (4.1)	0.41

**Table 4:** Identification of the main risk factor for chronicity (n = 484).

	Vignette A	Vignette B	Vignette C
Psychosocial, n(%)	464 (95.7)	80 (16.5)	320 (66)
Physical, n(%)	21 (4.3)	405 (83.5)	165 (34)

## Data Availability

All data derived from this study are presented in the text.

## Supplementary Materials

The following supporting information can be downloaded at: https://www.mdpi.com/article/10.3390/jcm12113865/s1, Supplementary File S1 contains the survey used in this study.
