# Supplementary material for: Consideration of Psychosocial Factors in Acute Low Back Pain by Physical Therapists
Source: J Clin Med. 2023 Jun 5;12(11):3865. doi: 10.3390/jcm12113865 (PMC10253485; doi:10.3390/jcm12113865)
Supplement: Supplementary file 1 [file jcm-12-03865-s001.zip › jcm-2305366-supplementary.pdf]

SURVEY ON THE DETECTION OF PSYCHOSOCIAL ASPECTS IN PATIENTS WITH LOW BACK PAIN

Below are two requirements you must meet to participate in this survey. IF YOU DO NOT FULFILL ANY OF THEM, you must submit it without completing the rest of the questions.

0.1 Are you a physiotherapist in the musculoskeletal field, being involved in the treatment of low back pain? Yes / No

0.2 Have you treated at least one case of acute low back pain in the last 12 months? Yes / No

If you agree to participate in this survey, please click the following box:

We invite you to participate in the research study "Consideration of psychosocial factors by physiotherapists in the management of patients with low back pain" promoted by Emilia Otero Ketterer, physiotherapist of Mutua Universal in Alcalá de Henares, whose objective is to analyze the detection of psychosocial factors in patients with low back pain, consisting of a survey directed to physiotherapists. This study is carried out following all national and international recommendations, having been evaluated and approved by the CEIC of the University of Alcalá. This is a voluntary survey, being able to withdraw whenever you want and without giving explanations or suffering any harm for it. Personal data (age and sex) will be collected along with professional data (years of experience, work setting and professional training) and a questionnaire of social and emotional intelligence. As this is a web-based survey, you will remain anonymous at all times and the answers will be automatically entered together with a code into an Excel document, which will be kept by the principal investigator. Therefore, we invite you to answer individually and as honestly as possible. Psychosocial factors have been shown to influence the chronicity of low back pain and there is controversy about the effectiveness of its detection, either through the clinical judgment of health professionals or through the use of formal detection tools. To find out how physiotherapists identify these factors, we have developed this web survey. The estimated response time is 20 minutes. It will start with a first part based on general questions addressed to you together with a social intelligence questionnaire and an emotional intelligence questionnaire. It will continue with a second part, consisting of three clinical cases (vignettes) of patients attending physiotherapy treatment for low back pain along with one question regarding the main risk for chronicity in each of them. Your participation will help us to know what aspects influence the identification of psychosocial factors in patients with low back pain. You will collaborate with the advancement of research and will allow your experience to be useful, in one way or another, for many people.

The processing, communication and transfer of your data will be done in accordance with the provisions of Law 15/1999 of 13 December on the protection of personal data and its regulations (RD 1720/2007). At any time, you may access, correct, or cancel your data.

We are available to answer any questions you may have about this study, in which case please do not hesitate to contact us at the email address provided below.

Thank you very much for your participation, Emilia Otero Ketterer emiliaoteroketterer@gmail.com

0.3 __ I agree to participate in the survey

General questions

A.1 Age: (Write the number of years, for example: 32)

A.2 Sex: Male / Female

A.3 Number of years of experience treating musculoskeletal disorders: (Write the number of years, e.g.: 8)

A.4 You practice your profession in:

a) Free practice

b) Mutual Health Insurance Companies collaborating with the Public Health Service

c) Public Health Service

A.5 Do you have specific postgraduate training in the psychosocial aspects of pain: Yes / No.

Social intelligence questionnaire:

For each of the following statements, indicate the degree to which each statement best defines you. (Labels on 1, 3, and 5. 1: Not at all, 3: Somewhat like me, 5: Yes! Very much like me).

		**NO! Not at all**		**Somewhat like me**		**Yes! Very much like me**
B1.	In conversation I am sensitive to even the slightest change in the facial expression of the person with whom I am conversing.	1	2	3	4	5
B2.	My powers of intuition are very good at understanding the emotions and motivations of others.	1	2	3	4	5
B3.	I often feel insecure around new people I don't know.	1	2	3	4	5
B4.	I am often able to read people's true emotions through their eyes.	1	2	3	4	5
B5.	I sometimes hurt others without realizing it.	1	2	3	4	5
B6.	I understand the feelings of others.	1	2	3	4	5
B7.	I find people unpredictable.	1	2	3	4	5
B8.	I can tell when I have said something inappropriate by reading in the other person's eyes.	1	2	3	4	5

		NO! Not at all		Somewhat like me		Yes! Very much like me
B9.	I have trouble changing my behavior and adapting to different people in different situations.	1	2	3	4	5
B10.	I can often understand what others mean through their expression, body language...etc.	1	2	3	4	5
B11.	I can predict the behavior of others.	1	2	3	4	5
B12.	Once I know what the situation calls for, it is easy for me to regulate my actions accordingly.	1	2	3	4	5
B13.	I have the ability to get along with strangers.	1	2	3	4	5
B14.	I often feel it is difficult to understand the decisions of others.	1	2	3	4	5
B15.	If someone is lying to me, I can usually tell from the start by the way they express themselves.	1	2	3	4	5
B16.	I can predict how others will react to my behavior.	1	2	3	4	5
B17.	I find it difficult to get along with other people.	1	2	3	4	5
B18.	It takes me quite a while to get to know other people well.	1	2	3	4	5
B19.	People often seem to be angry with me when I speak my mind.	1	2	3	4	5
B20.	I know how my actions will make others feel.	1	2	3	4	5
B21.	I can often understand what others are trying to accomplish without the need for them to tell me anything.	1	2	3	4	5
B22.	In social situations I have the ability to modify my behavior if I feel something requires it.	1	2	3	4	5
B23.	People often surprise me with the things they do.	1	2	3	4	5
B24.	I have found that I can adjust my behavior to whatever situation I find myself in.	1	2	3	4	5
B25.	I understand the desires of others.	1	2	3	4	5

		NO! Not at all		Somewhat like me		Yes! Very much like me
B26.	Even when it might be positive for me, I have difficulty putting on a good face.	1	2	3	4	5
B27.	I can usually tell when others think I am making a bad joke, even though they may laugh convincingly.	1	2	3	4	5
B28.	I fit in well in new social situations and meeting people for the first time.	1	2	3	4	5
B29.	When I feel that the image I am displaying does not work, I can easily try to change to another one that does.	1	2	3	4	5
B30.	I have the ability to control the way I approach people, depending on the impression I want them to have of me.	1	2	3	4	5
B31.	I frequently have trouble finding good topics to talk about.	1	2	3	4	5
B32.	Other people may get angry with me without my being able to figure out why.	1	2	3	4	5
B33.	I am good at dealing with new social situations and meeting people for the first time.	1	2	3	4	5
B34.	I am often surprised at other people's reactions to what I do.	1	2	3	4	5

Emotional intelligence questionnaire:

For each of the following statements, indicate the degree to which you agree with each statement (1: Do not agree at all, 2: Somewhat agree, 3: Fairly agree, 4: Strongly agree, 5: Strongly agree).

C1. I pay close attention to feelings.

I do not agree at all	Somewhat agree	Quite agree	Strongly agree	Totally agree

C2. I usually care a lot about how I feel.

I do not agree at all	Somewhat agree	Quite agree	Strongly agree	Totally agree

C3. I usually spend time thinking about my emotions.

I do not agree at all	Somewhat agree	Quite agree	Strongly agree	Totally agree

C4. I think my emotions and moods are worth paying attention to.

I do not agree at all	Somewhat agree	Quite agree	Strongly agree	Totally agree

C5. I let my feelings affect my thoughts.

I do not agree at all	Somewhat agree	Quite agree	Strongly agree	Totally agree

C6. I think about my mood constantly.

I do not agree at all	Somewhat agree	Quite agree	Strongly agree	Totally agree

C7. I often think about my feelings.

I do not agree at all	Somewhat agree	Quite agree	Strongly agree	Totally agree

C8. I pay close attention to how I feel.

I do not agree at all	Somewhat agree	Quite agree	Strongly agree	Totally agree

C9. I am clear about my feelings.

I do not agree at all	Somewhat agree	Quite agree	Strongly agree	Totally agree

C10. I can often define my feelings.

I do not agree at all	Somewhat agree	Quite agree	Strongly agree	Totally agree

C11. I almost always know how I feel.

I do not agree at all	Somewhat agree	Quite agree	Strongly agree	Totally agree

C12. I usually know my feelings about people.

I do not agree at all	Somewhat agree	Quite agree	Strongly agree	Totally agree

C13. I often realize my feelings in different situations.

I do not agree at all	Somewhat agree	Quite agree	Strongly agree	Totally agree

C14. I can always tell how I feel.

I do not agree at all	Somewhat agree	Quite agree	Strongly agree	Totally agree

C15. I can sometimes tell what my emotions are.

I do not agree at all	Somewhat agree	Quite agree	Strongly agree	Totally agree

C16. I can come to understand my feelings.

I do not agree at all	Somewhat agree	Quite agree	Strongly agree	Totally agree

C17. Although I sometimes feel sad, I tend to have an optimistic outlook.

I do not agree at all	Somewhat agree	Quite agree	Strongly agree	Totally agree

C18. Even when I feel bad, I try to think of pleasant things.

I do not agree at all	Somewhat agree	Quite agree	Strongly agree	Totally agree

C19. When I am sad, I think of all the pleasures in life.

I do not agree at all	Somewhat agree	Quite agree	Strongly agree	Totally agree

C20. I try to think positive thoughts even when I feel bad.

I do not agree at all	Somewhat agree	Quite agree	Strongly agree	Totally agree

C21. If I overthink things, complicating them, I try to calm down.

I do not agree at all	Somewhat agree	Quite agree	Strongly agree	Totally agree

C22. I take care to be in a good mood.

I do not agree at all	Somewhat agree	Quite agree	Strongly agree	Totally agree

C23. I have a lot of energy when I feel happy.

I do not agree at all	Somewhat agree	Quite agree	Strongly agree	Totally agree

C24. When I am angry, I try to change my mood.

I do not agree at all	Somewhat agree	Quite agree	Strongly agree	Totally agree

Clinical cases

Below you will find three clinical cases (vignettes) of patients with low back pain for which you will have to answer one question.

Vignette 1

47-year-old female clinic assistant who comes for physiotherapy treatment because she has had low back pain without pain radiating to the legs for three days. Since the beginning she has been able to do household chores but has not gone to work. Today she has noticed improvement after taking analgesics, reporting a pain rating of 4 out of 10. Her physical examination shows mild low back pain, with preserved mobility, strength, and reflexes in the lower extremities. She has a history of irritable bowel syndrome and anxiety and is currently on medication. She comments that she feels bad and very anxious since the onset of the pain. She does not recall previous episodes of low back pain. When asked if her work activity causes her low back pain, she denies it, but says that she is not very satisfied with her work.

Of the following types of factors, which is the one that contributes most to this patient's risk of chronicity:

- Physical
- Psychosocial

Vignette 2

55-year-old male plumber. He reports moderate lumbar pain referred to the posterior aspect of the right thigh when he sat up after having been bent over doing a repair. He has not been able to return to work since then. There has been no loss of strength. Currently and after 6 weeks of

evolution, he shows joint mobility with great limitation towards flexion and moderate towards extension, with minor right lumbar rotation. The straight leg raise test causes relevant pain with moderate limitation and shows no neurological deficits. She refers to previous episodes of low back pain, this being the worst of all, despite having taken analgesics and anti-inflammatory drugs. In general, he is in good health. The patient asks for advice on back exercises and postural hygiene, since he considers that he can improve the postures he adopts at work and is looking to resume ballroom dancing.

Of the following types of factors, which is the one that contributes most to this patient's risk of chronicity:

- Physical

- Psychosocial

Vignette 3

A 53-year-old woman who works in a clothing store comes with a 5-week history of low back pain radiating to the right leg after lifting at work and has been unable to work ever since. Her job involves constant bending and lifting. She has diffused numbness throughout the right leg if she bears weight for more than ten minutes, but no loss of strength. He reports a long history of intermittent low back pain that has previously taken up to 12 weeks to resolve. She is currently taking medication for hypertension and depression. She is having trouble performing household chores, walking, and driving. She also reports clear difficulty sleeping. Range of motion shows significant limitation to lumbar flexion, moderate limitation to extension and no loss of lateral movements. The straight leg raise test does not generate radiating pain but is slightly restricted. On examination there is no motor or sensory loss. She is referred by her company doctor.

Of the following types of factors, which is the one that contributes most to this patient's risk of chronicity:

- Physical

- Psychosocial

The survey has been successfully completed.

Thank you for your participation!
